# Influence of Ammonium Polyphosphate/Lignin Ratio on Thermal and Fire Behavior of Biobased Thermoplastic: The Case of Polyamide 11

**DOI:** 10.3390/ma12071146

**Published:** 2019-04-08

**Authors:** Aurélie Cayla, François Rault, Stéphane Giraud, Fabien Salaün, Rodolphe Sonnier, Loïc Dumazert

**Affiliations:** 1 ENSAIT, GEMTEX—Laboratoire de Génie et Matériaux Textiles, F-59000 Lille, France; aurelie.cayla@ensait.fr (A.C.); francois.rault@ensait.fr (F.R.); fabien.salaun@ensait.fr (F.S.); 2 IMT Mines d’Alès, Centre des Matériaux des Mines d’Alès–Pôle Matériaux Polymères Avancés, 30100 Alès, France; rodolphe.sonnier@mines-ales.fr (R.S.); loic.dumazert@mines-ales.fr (L.D.)

**Keywords:** lignin, polyamide 11, ammonium polyphosphate, thermal decomposition, fire reaction

## Abstract

Flame retardancy of polymers is a recurring obligation for many applications. The development trend of biobased materials is no exception to this rule, and solutions of flame retardants from agro-resources give an advantage. Lignin is produced as a waste by-product from some industries, and can be used in the intumescent formation development as a source of carbon combined with an acid source. In this study, the flame retardancy of polyamide 11 (PA) is carried out by extrusion with a kraft lignin (KL) and ammonium polyphosphate (AP). The study of the optimal ratio between the KL and the AP makes it possible to optimize the fire properties as well as to reduce the cost and facilitates the implementation of the blend by a melting process. The properties of thermal decomposition and the fire reaction have been studied by thermogravimetric analyzes, pyrolysis combustion flow calorimetry (PCFC) and vertical flame spread tests (UL94). KL permits a charring effect delaying thermal degradation and decreases by 66% the peak of heat release rate in comparison with raw PA. The fire reaction of the ternary blends is improved even if KL-AP association does not have a synergy effect. The 25/75 and 33/67 KL/AP ratios in PA give an intumescence behavior under flame exposure.

## 1. Introduction

Until now the plastic industry was governed by the use of petroleum resources. However, an evolution of the sector is underway. In the last few years, a transition has taken place between fully fossil-based polymers and fully-biobased polymers being replaced by partially biobased ones. Biobased polymers can be considered as macromolecular materials coming from biological resources and transformed by humans to be used in various activities in the form of massive sheets, films, or fibers. Biobased polyethylene (PE), polylactic acid (PLA), or even polyamide 11 (PA) derived from castor oil are some examples of novel thermoplastic polymers able to compete with conventional polymers. Nevertheless, in order to ensure their development and their use on the market, only emphasizing the environmental benefits for those polymers compared to those derived from petroleum resources is not enough. Indeed, these polymers must satisfy the same prerequisites as their predecessors. They have to reach the same criteria related to health, legislation, and the economy, as well as various performance criteria. The reduction of fire risks is one of the properties required and sometimes mandatory to access sectors such as transport and buildings. One of the challenges is then to guarantee the security of the proposed solutions while preserving as much as possible the bio-sourced aspect. Thus in recent years some of the research related to the flame retardancy of polymers has focused on the use of “green” flame retardant additives [1].

Different strategies exist and/or co-exist in order to fireproof petroleum-based polymers and can be transposed to the biobased ones. Two modes of actions, i.e., in the gas phase or condensed phase, may operate independently or together. Their mechanisms require radical inhibition or dilution of the combustion gas in the first mode, and endothermic degradation or dilution of the polymer, or even creation of a physical and/or thermal protective barrier, in the second mode. The latter approach refers mainly to the concept of intumescence and consequently to intumescent systems. An acidic source, a blowing or swelling agent, and a carbonizing agent are traditionally involved in the development of such systems that lead to the formation of an intumescent char (expanded carbonaceous barrier) upon heating. One of the most studied formulations is based on phosphorus-based additives able to release phosphoric acid, polyols as a carbon source and melamine as a blowing agent. These formulations can sometimes be limited to the use of only two components. Indeed, some components such as ammonium polyphosphate (AP) can act as an acid source and a swelling agent. Many studies have therefore been interested in developing fully (or at least partially) biobased intumescent formulations. In the case of biobased polymers, the use of natural additive is, even more so, an obvious solution to develop fire retardant formulation. It allows preservation of the original philosophy of sustainable development and the production of eco-friendly new high value-added polymers. Thus, for the first time, scientists have especially tried to substitute carbon sources traditionally used for biobased polymers. Formulations combining AP and alternative natural-based carbon sources such as starch [2,3,4], β-cyclodextrin [5,6,7], and chitosan [8] have been tested with one of the most investigated biobased polymers, i.e., PLA. Among the other natural-based compounds such as DNA, proteins, other saccharide-based compounds (sorbitol, xylitol, etc.), or derivatives from them (ex: isosorbide), biobased aromatic products, and in particular lignin, are of great interest to develop intumescent formulations. It is mentioned that its chemical structure (content of hydroxyl groups and aromatic structure) promotes the formation of a high char yield after decomposition and that its fire-retardant effect can be improved by associating other flame-retardant components [9]. Furthermore, lignin is an abundant co-product of the pulp and paper industry. Therefore, it can be considered as a waste of them and consequently provide a low-cost, eco-friendly alternative to petroleum-based carbon sources in intumescent systems for bioplastics. Its impact on the modification of the fire behavior of the polybutylene succinate [10,11], polylactic acid [2,12,13,14,15,16,17], or polyamide 11 [18,19,20] has been evaluated. 

The flame retardancy of a biobased polymer such as PLA is relatively well documented in the literature. Different scientists have worked on this issue by considering several strategies including the use of intumescent systems that may be or not bio-sourced. In contrast, the fire-retardant enhancement of other biobased thermoplastic by using the concept of intumescence, in particular in polyamide 11, has up to now more rarely been investigated. Levchick et al. [21] studied the fire behavior of polyamide 11 with AP. One of the main conclusions is that the yield of residue formed seems not to be significantly influenced by AP. To the best of our knowledge, our research group is the first to consider the development of an intumescent flame retardant system for polyamide 11 with a double target, i.e., i) push the “green” concept as far as possible, and ii) keep the additives content compatible with further textile fiber forming process. The purpose of this article is to study the combustion behavior of polyamide 11 containing intumescent system composed of lignin and AP. Blends of PA with different kraft lignin (KL)/AP ratio were prepared by melt extrusion. UL-94 and pyrolysis combustion flow calorimetry (PCFC) experiments evaluated the flame-retardant effectiveness of such a solution. Furthermore, the potential synergy between lignin and AP as well as their interactions with polyamide 11 were investigated. Even if the thermal behavior of the KL/AP powder mixtures is different with the KL/AP ratio, the PA blends with the same mixtures present a quite similar thermal and fire behavior without synergy compared to PA blends with only AP or KL.

## 2. Materials and Methods 

### 2.1. Materials and Processing

A biobased Polyamide 11 (PA), Rilsan® BMNO-TLD; *M*n = 17,000 g/mol, melt flow index (MFI) =14–20 g/10 min at 235 °C, supplied from Arkema (Colombes, France), was chosen as the polymer matrix. The lignin used as charring agent is a kraft lignin (KL) purchased from Sigma Aldrich (Darmstadt, Germany). Ammonium polyphosphate (AP) with the reference Exolit AP 422 was supplied by Clariant (Muttenz, Switzerland). The both additives consist of a thin powder with an averaged particle size of 39 µm and 15 µm respectively. All the materials were dried at 80 °C for 24 h before any use.

### 2.2. Blends Preparation

The compositions of all the samples are listed in Table 1. In order to understand the influence of coupled additives on PA, the thermal decomposition of simple powder mixtures of both additives was assessed. Blends with PA were prepared with a suitable quantity of polymer and additives (lignin and/or ammonium polyphosphate). In order to obtain homogeneous pellets, each premix was extruded in a co-rotating, intermeshing twin screw extruder Thermo Haake (diameter of screw = 16 mm, L/D ratio = 25) from Thermo Fisher Scientific (Waltham, MA, USA). The rotating speed was maintained at 100 rpm. The five heating zones of the extruder were set at temperatures 170, 190, 200, 220, and 220 °C respectively to keep the highest fluidity without damaging the components. The different formulations extruded (with or without fillers) were then cooled under ambient air and pelletized. The pellets obtained for each formulation were transformed in plates of 100 × 13 × 3 mm^3^ (for UL94 tests) using a heat press from Dolouets (Soustons, France) at 220 °C under 50 bars.

### 2.3. Thermal Decomposition

Thermogravimetric analyses were performed using a TA instruments thermal analyzer model number 2050 (New Castle, DE, USA) to study the thermal stability of blends. The sample of 6 ± 1 mg was placed in an open platinum crucible, and an empty platinum crucible was used as the reference. Dynamic runs were carried out from room temperature to 600 or 800 °C, at a heating rate of 10 °C/min in a 50 mL/min flow of nitrogen. Thermogravimetric curves (TG) curves were recorded from experiments, and Derivative thermogravimetric (DTG) curves were obtained from TA universal data analysis software for all the samples. The decomposition parameters, such as the temperature at 5% and 50% weight loss (*T*_5%_ and *T*_50%_), and residue at 600 or 800 °C were obtained from the TG curve. Furthermore, the maximum mass loss rate (*R*_max_) and the corresponding temperature (*T*_max_) for each main degradation step were obtained from DTG curves.

The residual mass difference curves were plotted in order to determine the increase or decrease in the thermal stability of the blends with the subsequent interaction between components. The residual mass difference curves were computed for the powder additive mixtures and composites samples, and correspond to the residual mass difference between the experimental and theoretical TG curves Equation (1).

(1) $$\Delta (M(T))=M_{\exp}(T)-M_{theo}(T),$$

where, Δ(M(T)) is a residual mass difference, $M_{\exp}(T)$ is the experimental residual mass of blends (variation with temperature T), $M_{theo}(T)$ is the theoretical residual mass of the same composition computed by a linear combination between the experimental masses of each components. The residual mass difference of the blend at the temperature T permits to give an indication on thermal stability of the blend regarding the whole thermal history until the temperature T.

### 2.4. Fire Reaction

The flammability was evaluated on sample bars (125 × 12.5 × 3 mm^3^) by vertical flame spread tests according to IEC 60695-11-10 [22], also known as UL 94 burning flame test and used for plastic materials. This test is aimed at assessing the material capability to extinguish a flame. Materials were classified from their burning rate, time to flame out and dripping during burning.

We also employed pyrolysis combustion flow calorimetry (PCFC) (Fire Testing Technology, East Grinstead, UK). This technique was described by Lyon [23]. Sample amount of some milligrams is pyrolyzed with a heating ramp (1 K/s) up to 750 °C under nitrogen flow. The gases released during the pyrolysis are removed in an oven at 900 °C in the presence of an 80/20 N_2_/O_2_ mixture. In these conditions, the total combustion of these gases takes place. According to Huggett’s relation (1 kg of consumed O_2_ corresponds to 13.1 MJ of released energy), the measurement of oxygen consumption by PCFC permits to calculate the heat release rate. Three tests were carried out on each formulation, and the results averaged. According to this analytical method, usual parameters were evaluated, i.e., the peak of heat release rate (pHRR) and its temperature, total heat release (THR), char residue and heat of complete combustion (Δh) which is calculated by the division of the THR on mass loss fraction.

## 3. Results and Discussion

### 3.1. Thermal Degradation

**Figure 1:**
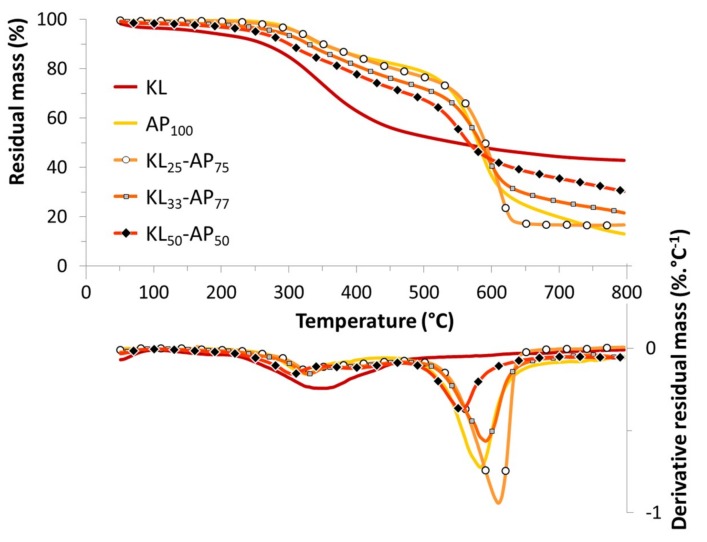
TG and DTG curves of KL, AP, and their blends.

Lignin is composed of many components having different decomposition pathways leading its thermal degradation following a complex process with consecutive reactions [24]. Thus, lignin decomposes slower over a broad temperature range from 200 to 500 °C, and the DTG curve shows a wide and flat peak with a gentle slope line (Figure 1), due to the presence of various oxygen functional groups in its structure. Each group has different thermal stability, and therefore their scission occurs at different temperatures. During the thermal decomposition, the cleavages of the lignin functional groups lead to the formation of low molecular weight products until the chemical rearrangement of the backbone at a higher temperature with the formation of a significant char and the release of volatile products. Up to 200 °C, lignin has an initial weight loss equal to around 5%, mainly due to moisture release and volatile impurities. Above this temperature, the compound undergoes a two steps degradation process under an inert atmosphere. From 200 to 500 °C, heated up by 10 °C·min^−1^, lignin decomposes very slowly, since the max rate of degradation at 355 °C is only about 0.2433 %·°C^−1^, losing only 48 wt. % of its initial mass below 500 °C (Table 2). During this main degradation step, the cleavage of the aryl-ether linkages results in the formation of aromatic hydrocarbons, phenolic, hydroxyphenolics, and guaiacyl/syringyl-type compounds according to Rodrigues et al. [25]. Thereafter, at the end of the pyrolysis phenols groups are transformed into pyrocatechols [26]. The degradation rate slightly decreases to 0.03 %·°C^−1^ during the second step, with the formation of a residue of about 43 wt. % at 800 °C due to the formation of polycyclic aromatic hydrocarbons.

The thermal decomposition of ammonium polyphosphate occurs in two-steps mechanism in nitrogen conditions [27]. The first degradation step from 200 to 450 °C, with 18% of weight loss, involves the water and ammonia releases as the gaseous products. Maximum degradation peak was observed at approximately 327 °C. The water elimination further induces the formation of phosphoric acid coupled to a cross-linking mechanism to lead the formation of polyphosphoric acid. The second degradation step from 450 to 700 °C is related to the dehydration and the fragmentation of polyphosphoric acid to form phosphorus oxides. In this temperature range, this material undergoes a sharp weight loss (69 wt. %) with the formation of a stable residue (13 wt. %) upon heating up to 800 °C.

The presence of ammonium polyphosphate changed the thermal behavior of lignin (Figure 1 and Table 2). Regardless of the ratio, *T*_5%_ and *T*_50%_ were found to be increased. Compared with pure KL, the higher initial degradation temperature is due to the higher thermal stability of AP, while the much higher *T*_50%_ (improvement of thermal stability) is mainly attributed to a much higher char residue. Furthermore, the thermal degradation occurs in two consecutive steps in the same temperature ranges than the AP one for the samples KL_25_-AP_75_ and KL_33_-AP_67_. According to the DTG curves, the maximal rate of weight loss for two-step degradation is at 328 and 593 °C, 333 and 611 °C for KL_33_-AP_67_ and KL_25_-AP_75_, respectively. The decomposition shift to a higher temperature may be attributed to the increasing content of AP. It can also be noticed the presence of an intermediate step for the sample labeled KL_50_-AP_50_ between 350 and 470°C. The first step degradation of KL-AP blends occurs at a lower temperature compared to KL, whereas the second step is shifted towards higher temperature compared to AP, except for the sample labeled KL_50_-AP_50_. Above 200°C, the mass loss rates decrease in comparison to that for KL leading to stabilization until the second degradation step. Thus, *R*_max_ is indeed reduced from 0.24 to 0.13 or 0.15 wt. %·°C^−1^ under pyrolytic conditions. It appears that the adding of AP induces a slight thermal destabilization in the first step decomposition because phosphoric acid molecules catalyzed dehydration of lignin. For the second or third (sample for KL_50_-AP_50_) degradation step, *R*_max_ values are found to be lower than for AP when AP content is less than 75% in weight, and higher for this sample. At high temperature, the residue amount increases from 16.5 to 33 wt. % as the AP content decreases. 

**Figure 2:**
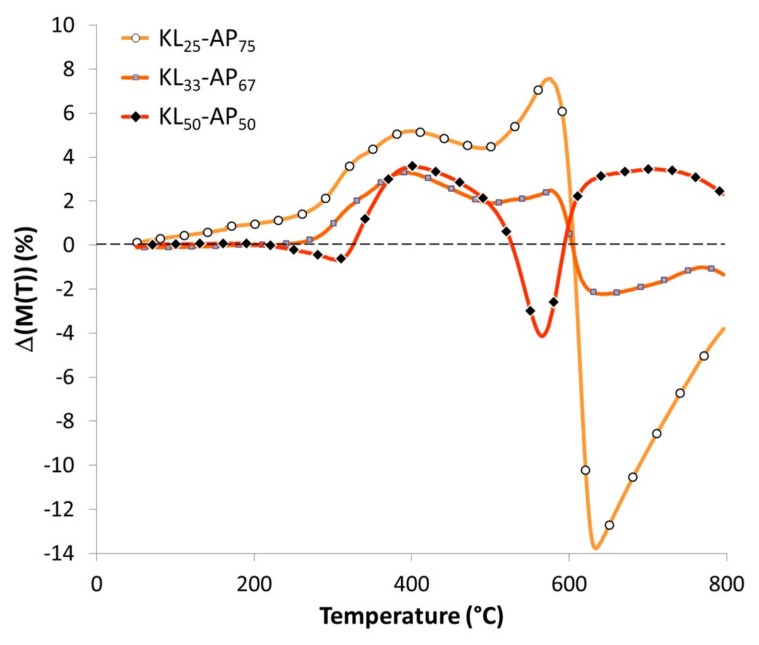
Curves of residual mass loss difference for KL-AP powder blends.

Figure 2 allows to point out the interactions between the decomposition products of KL and AP during thermal degradation of the KL-AP mixtures. The KL_25_-AP_75_ and KL_33_-AP_67_ blends present until 600 °C a thermal stabilization phase where the residual mass of the blend is higher than the addition of the residual mass of KL and AP. This positive interaction between the decomposition products of KL and AP has two local maxima at 390 °C and 570 °C. After 600 °C, both blends are thermally destabilized with minima at 630 °C. The positive and negative interactions are more amplified for KL_25_-AP_75_ (maximum +7.5% / minimum −13.7%) than for KL_33_-AP_67_ (maximum +3.3%/minimum −2.2%). The KL_50_-AP_50_ blend also has a maximum positive interaction +3.5% at 390°C, but between 530 and 600 °C, the blend presents a thermal destabilization with −4% minimum. Above 600 °C, the decomposition products interact again positively with +3.5% maximum at 710 °C. Thus, smaller AP contents lead to a higher charring effect with better thermal stabilization.

**Figure 3:**
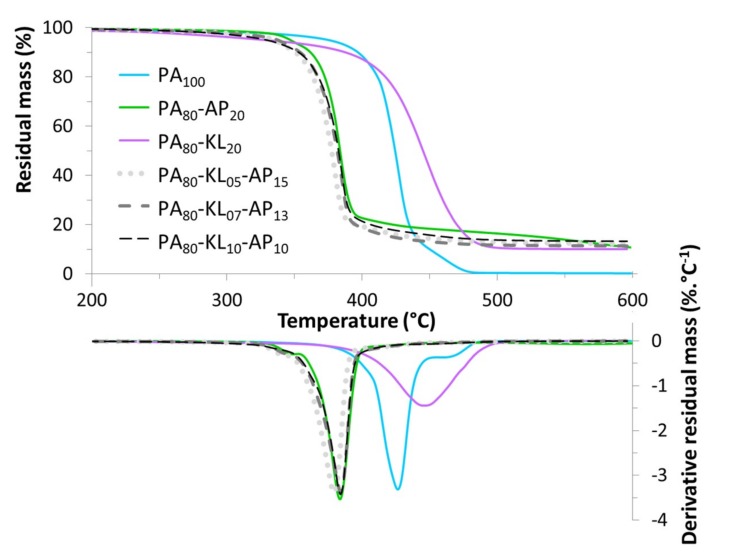
TG and DTG curves of PA and PA blends with AP and KL under nitrogen atmosphere.

**Figure 4:**
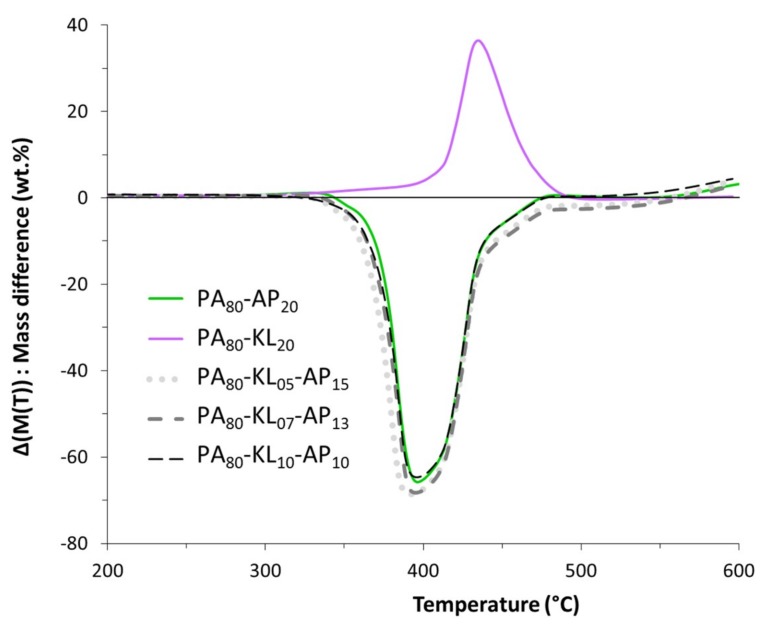
Curves of residual mass loss difference for PA blends with AP and KL.

TG and DTG curves of PA and PA/lignin and/or ammonium polyphosphate blends under N_2_ atmosphere are shown in Figure 3. It can be observed that the neat PA shows a two-step thermal degradation in the range of 350–500°C with no char residue left at 600 °C. The primary step is located around 430 °C, whereas the second and minor step occurs over 450 °C due to the decomposition of cross-linked structures obtained during heating, as a shoulder in the DTG curve suggests [21]. For PA with 20% of AP, the onset temperature and the temperature at the maximum rate of weight loss (Table 3) are shifted toward low temperature by respectively 30 and 40 °C compared to raw PA, due to the early decomposition of AP. The polyphosphoric acid released from the ammonium polyphosphate decomposition reacted with the amide bond to form intermediate phosphate ester bonds, leading to a decrease at *T*_50%_ of PA by 40 °C. A substantial thermal destabilization for the blend PA_80_-AP_20_ is also observed on the differential mass curve (Figure 4) between 350 and 500 °C. The phosphate ester bonds decomposed at a higher temperature to promote the formation of char, it can be observed that the obtained residue at 600 °C is slightly higher than the theoretical values (Table 3).

The presence of lignin at 20% in PA is responsible for the decrease of *T*_5%_ by 50 °C, and an increase of *T*_50%_ and *T*_max_ about 20 °C. Furthermore, the thermal degradation occurs in a single, broader stage. The decrease of *T*_5%_ may be attributed to a rapid mass loss rate of KL at a lower temperature region. The difference between TG curves of raw and filled polymer (Figure 4) shows a noticeable thermal stabilization between 400 and 500 °C. So KL permits a charring effect delaying thermal degradation of the blend in comparison with raw PA. However, above 500 °C the residual mass difference of the blend is 0 and the residue amounts correspond to the KL content after its degradation. Thus, it can be concluded that there is no interaction between decomposition products of PA and KL.

The mass loss behavior of PA_80_-KL_05_-AP_15_, PA_80_-KL_07_-AP_13_, and PA_80_-KL_10_-AP_10_ samples (Figure 3) are almost similar, and their DTG maxima are observed at 379 and 384 °C, respectively. The influence of AP in the thermal degradation of the ternary blends is predominant since their TG curve is closed to the TG curve of PA_80_-AP_20_. The onset temperature, *T*_5%_, of the ternary blends are found to be decreased compared to raw PA and also PA-AP samples, by 40 to 50 °C and 11 to 18 °C, respectively. Their decomposition temperatures (*T*_max_) are decreased compared to that of raw PA, whereas the maximum rates of degradation are found to be slightly increased for the samples PA_80_-KL_07_-AP_13_ and PA_80_-KL_10_-AP_10_, and more significantly for the PA_80_-KL_05_-AP_15_ one. Therefore, the presence of AP has a catalyzing effect on the degradation of PA, which is stronger in presence of KL in the initial step of degradation. Furthermore, the TG curve of KL shows that 7% of KL decomposes at 220 °C, i.e., at processing temperature to prepare PA-KL-AP blends. Thus, the decrease of *T*_5%_ of these blends by 30 to 40 °C is due to early decomposition of KL and AP. As for mass loss difference of PA_80_-AP_20_ sample, the mass loss difference of the three ternary blends (Figure 4) presents a critical thermal destabilization period from 350 and 500 °C. It can also be noticed that the thermal degradation of the three ternary blends seems to be irrelevant of the AP to KL weight ratio used in this study, since the change trends of TG curves between all samples are similar, even if there are still some differences. The initial decomposition temperature of PA_80_-KL_10_-AP_10_ is lower than the two other samples ones. Besides, for a sufficient AP content, when samples begin to degrade, the charring aromatic radicals coming from KL reduce the polymer degradation rate increasing the composite thermal degradation temperature by 5 °C. On the other hand, the residue left at 600 °C for PA_80_-KL_05_-AP_15_, PA_80_-KL_07_-AP_13_, and PA_80_-KL_10_-AP_10_ are about 12.3, 11.3, and 13.3, respectively, suggesting higher charring than PA-KL and PA-AP samples. Therefore, the increase in the amount of residue may be owing to the formation of more stable carbonaceous char. Indeed, the char yield for the three ternary blends at 600 °C (Table 3) is higher than the sum of the individual contributions of each component. Thus, more effective carbonizing and cross-linking reactions take place during the PA degradation on the addition of AP with KL in comparison to KL or AP solely. From the above results, It seems that the blend with the 50:50 weight ratio of KL and AP is better than the two other ones (25:75 and 33:67) in improving the charring of the PA composite.

### 3.2. Flammability Behavior

**Figure 5:**
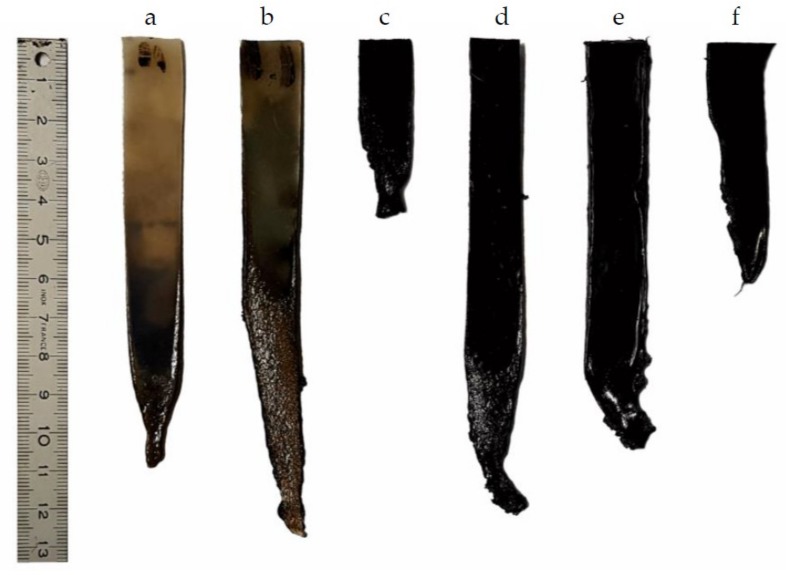
Pictures of PA blends specimen left after UL 94 vertical flame test. (**a**) PA; (**b**) PA_80_-AP_20_; (**c**) PA_80_-KL_20_; (**d**) PA_80_-KL_05_-AP_15_; (**e**) PA_80_-KL_07_-AP13; (**f**) PA_80_-KL_10_-AP_10_.

The results of the UL94 tests for PA and the blends are summarized in Table 4, and the typical pictures of the specimens left after the tests are shown in Figure 5. 

Dripping and cotton ignition were observed in all the blends. Just after the flame exposure, PA presents a significant dripping of burning materials. As already described in literature for some thermoplastic polymers like Polyamide 6 [28], the dripping is so consequent that the flame spread is limited and so the remaining preserved material is noticeable. The presence of AP or/and KL does not give significant improvement in the burning behavior of PA. With 20% of AP, we can observe during flame exposition a low charring effect with some crackling due to gas action of AP (ammonia and water release). Contrary to raw PA, the combustion is maintained during 10 s more in total after removing of the flame. The flame behavior of thermoplastic polymers in the vertical direction is complex, not only the mechanisms of thermal degradation have a determining role, but the viscoelastic properties in the molten state are also crucial [29]. The viscosity of molten PA_80_-AP_20_ blend is higher than for PA (Table S1). This difference of viscosity could be not favorable to PA_80_-AP_20_ blend from a flame spread point of view. The combustion stopped finally by dripping of burning materials. The combustion time for PA_80_-KL_20_ after the first flame exposure is short due to a rapid dripping of burning material, but after the 2^nd^ exposure, the dripping is slowed down, and a small flame spread during almost 15 s destroyed two-thirds of the samples. The best results are obtained with PA_80_-KL_05_-AP_15_ and PA_80_-KL_07_-AP_13_ which show a significant flame behavior improvement. A charring effect with some intumescence is observable during flame exposure, and the material is self-extinguishing after the first exposure. During the second exposure, some burning material drips but the samples kept finally about 80% of the initial mass. In the case of PA_80_-KL_10_-AP_10_, the charring effect is lower than in the case of the two other ternary blends, and no intumescence is observed, allowing the flame to spread on almost the entire sample.

### 3.3. Combustion Behavior

**Figure 6:**
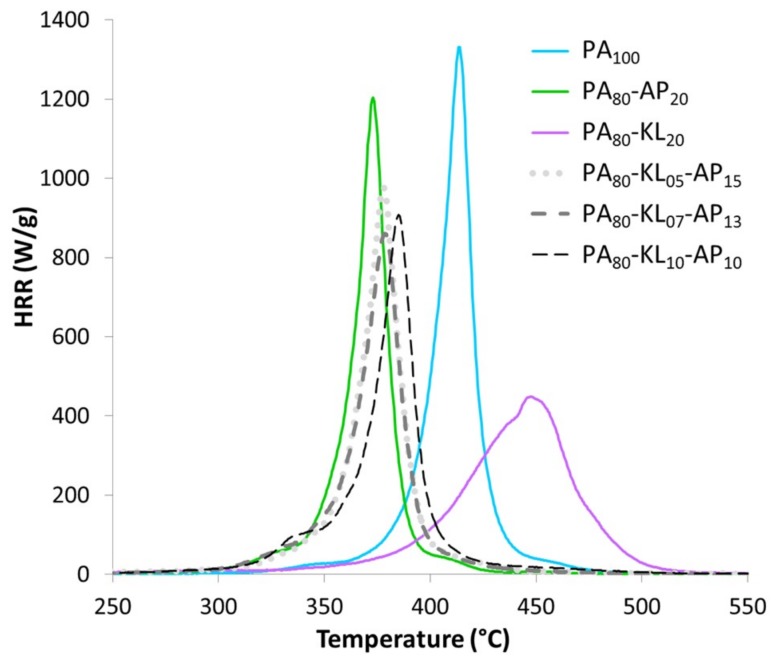
Curves of heat release rate (HRR) from PCFC experiments for PA blends with AP and KL.

The PCFC experiments provide access to the heat release rate from the complete combustion of fuel released during the anaerobic pyrolysis of the material. Table 5 presents for each PA blend the peak of heat release rate with its temperature, the total heat release, the char residue, and the heat of complete combustion. The Figure 6 shows the curves of HRR. HRR curves are in good agreement with DTG curves in Figure 3. Indeed, pyrolysis conditions are similar in PCFC and TGA in nitrogen.

Among the PA blends with KL and/or AP, the PA_80_-KL_20_ blend presents the best behavior combustion since the pHRR is decreased by 66% and shifted by around +40 °C in comparison of raw PA. The PCFC results for PA_80_-KL_20_ blend are correlated to the fact that the thermal degradation with 20% of KL is delayed, and slowed down compared to raw PA. However, the residue content for the PA_80_-KL_20_ blend remains quite low, and so the total heat released of PA_80_-KL_20_ is only slightly decreased (13%) compared to the THR of raw PA. The decrease of the Δh for PA_80_-KL_20_ blend (31.7 kJ/g) is limited (33.6 kJ/g for raw PA) and can be assigned to the replacement of a fraction of PA by KL having a low heat of combustion (around 10 kJ/g) [30]. Given PCFC results, the PA_80_-AP_20_ shows the lowest improvement of flame retardant effect. The HRR peak, the THR and the Δh of PA_80_-AP_20_ are closed to the raw PA values. Moreover, since PA_80_-AP_20_ has lower thermal stability than PA, the pHRR for PA_80_-AP_20_ is 30 °C below. The PCFC results for the three ternary blends are slightly better than for PA_80_-AP_20_ blend. Once again, residue content is slightly enhanced compared to binary blends. Therefore THR is reduced. Nevertheless, the destabilization due to AP leads to a pHRR at low temperature (around 30 °C below than that of PA, and the pHRR remains very high even if it is slightly reduced compared to raw PA and PA_80_-AP_20_.

## 4. Conclusions

The thermogravimetric analyses of the kraft lignin/ammonium polyphosphate powder mixtures, respectively charring agent and acidic source for potential flame retardant intumescent formulation point out noticeable interactions between both components whatever the ratio of the mixture. However, the KL-AP ratio influences these interactions. The curves of residual mass loss difference for KL-AP powder blends reveal a positive interaction between around 300 and 500 °C. However, unlike the KL_25_-AP_75_ and KL_33_-AP_67_ mixtures, only the KL_50_-AP_50_ mixture which has the highest residue at 800 °C keeps a positive interaction above 600 °C. The influence of KL-AP ratio on the thermal decomposition of the PA-KL-AP blends is low. The thermal degradations of the different ternary blends are similar and close to the decomposition of the PA-AP blend. AP finally dominates the degradation of the ternary blends. On the other hand, the presence of 20% of KL alone in PA has a positive effect on the thermal degradation with a delay in the main PA degradation step. The analyze of the fire behavior for the different PA blends by PCFC indicates that the fire retardant capacity of the ternary blends is intermediate considering the peak of HRR between these of the PA-AP and PA-KL blends. If the PA-KL blend shows the smallest peak of HRR, it is probably due mainly to the low combustion heat of the lignin. The KL-PA blend also presents the worst result with UL94 flammability test and, contrary to the PCFC test where the fire retardant effect by intumescence are not favored, the ternary blends present the best results even if they still V2 ranking due to creep phenomenon. For this test, the KL-AP ratio seems to have of influence since the formation of an efficiently expanded char is observed only for the PA_80_-KL_05_-AP_15_ and PA_80_-KL_07_-AP_13_ blends. In the case of PA_80_-KL_10_-AP_10_ blend, the flame behavior becomes more similar to the PA_80_-KL_20_ blend. Even if interactions between AP and KL exist, once both components are dispersed in PA, these interactions develop less. That is why the ternary blends do not present synergy on fire retardant aspect. Nevertheless, the ratio KL-AP affects the PA fire behavior, and given all the results, the PA_80_-KL_07_-AP_13_ blend seems to have the best fire retardant. 

## Figures and Tables

**Table 1:** Blend formulations: kraft lignin (KL)-ammonium polyphosphate (AP) powder mix and polyamide 11 (PA) composites.

Sample	PA (wt. %)	KL (wt. %)	AP (wt. %)
KL_25_-AP_75_	-	25	75
KL_33_-AP_67_	-	33	67
KL_50_-AP_50_	-	50	50
PA_100_	100	-	-
PA_80_-KL_20_	80	20	-
PA_80_-AP_20_	80	-	20
PA_80_-KL_05_-AP_15_	80	5	15
PA_80_-KL_07_-AP_13_	80	7	13
PA_80_-KL_10_-AP_10_	80	10	10

**Table 2:** TGA results of lignin, ammonium polyphosphate and their respective blends under nitrogen atmosphere.

Samples	*T*_5%_ (°C)	*T*_50%_ (°C)	*T*_max_ (°C) & *R*_max_ (%·°C^−1^)			Residue (%)^1^
			Step I	Step II	Step III	800 °C
KL	185	551	355 0.2433	-	-	43.0
AP	311	586	327 0.1345	583 0.7235	-	13.0
KL_50_-AP_50_	260	570	305 0.1571	386 0.1210	557 0.3696	33.0 (28.0)
KL_33_-AP_67_	280	578	328 0.1557	593 0.5613	-	23.7 (23.0)
KL_25_-AP_75_	314	590	333 0.1487	611 0.9401	-	16.5 (20.5)

^1^ in brackets calculated values based on additive behavior.

**Table 3:** TGA results of PA and blends with lignin and/or ammonium polyphosphate under nitrogen atmosphere.

Samples	*T*_5%_ (°C)	*T*_50%_ (°C)	*T*_max_ (°C) & *R*_max_ (%·°C^−1^)			Residue (%)^1^
			Step I	Step II	Step III	600 °C
PA_100_	375	424	426 3.320	463 0.361	-	0.2
PA_80_-AP_20_	347	383	384 3.485	-	-	10.7 (7.3)
PA_80_-KL_20_	325	445	445 1.443	-	-	10.0 (9.5)
PA_80_-KL_05_-AP_15_	335	377	379 3.888	-	-	12.3 (8.4)
PA_80_-KL_07_-AP_13_	336	381	384 3.381	-	-	11.3 (8.1)
PA_80_-KL_10_-AP_10_	329	382	384 3.431	-	-	13.3 (8.6)

^1^ in brackets calculated values based on additive behavior.

**Table 4:** UL94 vertical flame spread test data for PA and its blends.

Samples	1st Flame t_1_ (s)	2nd Flame t_2_ (s)	Combustion Time (t_1_+t_2_)	Cotton Ignition	Rating	Mass Loss (%)
PA_100_	1.6 ± 0.4	2.4 ± 0.6	4.0 ± 1.0	Yes	V2	33.7 ± 10.9
PA_80_-AP_20_	8.9 ± 5.9	4.8 ± 1.6	13.7 ± 5.7	Yes	V2	34.7 ± 4.9
PA_80_-KL_20_	1.1 ± 0.1	14.4 ± 11.3	15.5 ± 11.5	Yes	V2	64.1 ± 3.3
PA_80_-KL_05_-AP_15_	1.9 ± 0.9	2.7 ± 1.4	4.6 ± 1.9	Yes	V2	21.4 ± 3.3
PA_80_-KL_07_-AP_13_	3.0 ± 2.2	7.0 ± 1.9	10.0 ± 1.2	Yes	V2	19.1 ± 4.5
PA_80_-KL_10_-AP_10_	6.6 ± 0.7	22.8 ± 5.9	29.4 ± 6.5	Yes	V2	81.5 ± 16.6

**Table 5:** Pyrolysis combustion flow calorimetry (PCFC) data for PA and its blends. pHRR: peak of heat release rate; THR: total heat release; Δh: heat of complete combustion.

Samples	pHRR (W/g)	pHRR Reduction (%)	pHRR Temperature (°C)	THR (KJ/g)	THR Reduction (%)	Residue (%)	Δh (KJ/g)
PA_100_	1293 ± 39	-	413 ± 2	32.4 ± 0.1	-	3.2 ± 0.3	33.6 ± 0.4
PA_80_-AP_20_	1108 ± 92	14	382 ± 3	29.3 ± 0.4	10	11.4 ± 0.0	33.0 ± 0.4
PA_80_-KL_20_	442 ± 5	66	451 ± 2	28.3 ± 0.1	13	11.0 ± 0.2	31.7 ± 0.1
PA_80_-KL_05_-AP_15_	993 ± 30	23	380 ± 1	27.9 ± 0.2	14	14.4 ± 0.4	32.6 ± 0.4
PA_80_-KL_07_-AP_13_	908 ± 47	30	379 ± 0	26.8 ± 0.5	17	13.9 ± 0.1	31.1 ± 0.6
PA_80_-KL_10_-AP_10_	924 ± 19	29	383 ± 2	26.9 ± 0.9	17	12.1 ± 0.3	30.5 ± 0.4

## Supplementary Materials

The following are available online at https://www.mdpi.com/1996-1944/12/7/1146/s1, Table S1: Melting Flow Index for PA and its blends.
