# Supplementary material for: Influence of Ammonium Polyphosphate/Lignin Ratio on Thermal and Fire Behavior of Biobased Thermoplastic: The Case of Polyamide 11
Source: Materials (Basel). 2019 Apr 8;12(7):1146. doi: 10.3390/ma12071146 (PMC6479977; doi:10.3390/ma12071146)
Supplement: Supplementary file 1 [file materials-12-01146-s001.pdf]

materials

# Supporting Information

# Influence of Ammonium Polyphosphate / Lignin Ratio on Thermal and Fire Behavior of Biobased Thermoplastic: The Case of Polyamide 11

**Aurélie Cayla [1], François Rault [1], Stéphane Giraud [1,*], Fabien Salaün [1], Rodolphe Sonnier [2] and Loïc Dumazert [2]**

[1] ENSAIT, GEMTEX – Laboratoire de Génie et Matériaux Textiles, F-59000 Lille, France; aurelie.cayla@ensait.fr (A.C.); francois.rault@ensait.fr (F.R.); fabien.salaun@ensait.fr (F.S.)
[2] IMT Mines d'Alès, Centre des Matériaux des Mines d'Alès–Pôle Matériaux Polymères Avancés, Alès, France; rodolphe.sonnier@mines-ales.fr (R.S.); loic.dumazert@mines-ales.fr (L.D.)
* Correspondence: stephane.giraud@ensait.fr; Tel.: +33-320-256-464

The Melt Flow Tester from Thermo Haake (Thermo Fisher Scientific; Waltham, MA, USA) was used for Melt Flow Index (MFI) measurement to analyze spinnability and determine the spinning temperature condition. According to the standard ASTM D1238 [1], the piston and the dried material (7 g per measurement) were pre-heated for 4 and 3 min, respectively. This procedure was carried out twice for each blend at 200 °C under a load of 2.16 kg.

**Table S1.** Melting Flow Index for PA and its blends.

| Samples | MFI (200 °C, 2.16 kg) |
|---|---|
| $PA_{100}$ | 23.2 ± 0.5 |
| $PA_{80}$-$AP_{20}$ | 5.3 ± 0.8 |
| $PA_{80}$-$KL_{20}$ | 9.0 ± 1.0 |
| $PA_{80}$-$KL_{05}$-$AP_{15}$ | 5.6 ± 0.8 |
| $PA_{80}$-$KL_{07}$-$AP_{13}$ | 6.1 ± 0.9 |
| $PA_{80}$-$KL_{10}$-$AP_{10}$ | 6.7 ± 0.9 |

## Reference

1. ASTM. *ASTM D1238-13: Standard test method for melt flow rates of thermoplastics by extrusion plastomete*. ASTM International: West Conshohocken, PA, USA, 2013.
